# Genome-wide identification and expression analysis of Raffinose synthetase family in cotton

**DOI:** 10.1186/s12859-021-04276-4

**Published:** 2021-06-29

**Authors:** Ruifeng Cui, Xiaoge Wang, Waqar Afzal Malik, Xuke Lu, Xiugui Chen, Delong Wang, Junjuan Wang, Shuai Wang, Chao Chen, Lixue Guo, Quanjia Chen, Wuwei Ye

**Affiliations:** 1 College of Agriculture / Xinjiang Agricultural University / Xinjiang Research Base, State Key Laboratory of Cotton Biology, Urumqi, 830052 Xinjiang China; 2 State Key Laboratory of Cotton Biology / Key Laboratory for Cotton Genetic Improvement, Ministry of Agriculture / Institute of Cotton Research of Chinese Academy of Agricultural Sciences, Anyang, 455000 Henan China; 3 College of Biology and Food Engineering, Anyang Institute of Technology, Anyang, 455000 Henan China

**Keywords:** *RAFS*, *Gossypium* species, Gene co-expression network, Abiotic stresses, Gene duplication

## Abstract

**Background:**

The Raffinose synthetase (*RAFS)* genes superfamily is critical for the synthesis of raffinose, which accumulates in plant leaves under abiotic stress. However, it remains unclear whether *RAFS* contributes to resistance to abiotic stress in plants, specifically in the *Gossypium* species.

**Results:**

In this study, we identified 74 *RAFS* genes from *G. hirsutum*, *G. barbadense*, *G. arboreum* and *G. raimondii* by using a series of bioinformatic methods. Phylogenetic analysis showed that the *RAFS* gene family in the four *Gossypium* species could be divided into four major clades; the relatively uniform distribution of the gene number in each species ranged from 12 to 25 based on species ploidy, most likely resulting from an ancient whole-genome polyploidization. Gene motif analysis showed that the *RAFS* gene structure was relatively conservative. Promoter analysis for *cis*-regulatory elements showed that some *RAFS* genes might be regulated by gibberellins and abscisic acid, which might influence their expression levels. Moreover, we further examined the functions of *RAFS* under cold, heat, salt and drought stress conditions, based on the expression profile and co-expression network of *RAFS* genes in *Gossypium* species. Transcriptome analysis suggested that *RAFS* genes in clade III are highly expressed in organs such as seed, root, cotyledon, ovule and fiber, and under abiotic stress in particular, indicating the involvement of genes belonging to clade III in resistance to abiotic stress. Gene co-expressed network analysis showed that *GhRFS2A-GhRFS6A*, *GhRFS6D*, *GhRFS7D* and *GhRFS8A-GhRFS11A* were key genes, with high expression levels under salt, drought, cold and heat stress.

**Conclusion:**

The findings may provide insights into the evolutionary relationships and expression patterns of *RAFS* genes in *Gossypium* species and a theoretical basis for the identification of stress resistance materials in cotton.

**Supplementary Information:**

The online version contains supplementary material available at 10.1186/s12859-021-04276-4.

## Background

Raffinose family oligosaccharides (RFOs) accumulating in leaves during plant development are thought to play a vital role in the stress tolerance of plants [1]. Raffinose synthetase (RAFS) and Galactinol synthetase (GOLS) are two major watersoluble carbohydrates from RFOs that are responsible for raffinose biosynthesis in plants [2, 3]. Raffinose, the smallest member of RFOs, is widely found in the leaves, roots, seeds and tubers of plants [1, 4]. Studies have indicated that raffinose synthetase has homology with stachyose synthetase [5]. Heterologous expression of the *RAFS* gene in peas has shown that *RAFS* is a transglycosidase similar to galactosidase in structure and biochemical properties [6]. *RAFS* catalyzes the synthesis of raffinose, with galactinol as a galactosyl donor and sucrose as an acceptor [2]. The function and expression profile of *GOLS* under abiotic stress have been well clarified in many plant species. Some *GOLS* are expressed under the induction of abiotic stress, such as heat shock, drought, osmotic shock and salinity [7, 8]. Over-expression of *GOLS* has been found to increase the content of galactinol and raffinose and then improve abiotic stress tolerance [7, 9, 10]. Compared with synthetase *GOLS,* there have been few studies on the plant *RAFS* gene. The earliest reported *RAFS* gene was cloned from peas [1, 11], and later, the *RAFS* genes were cloned [2, 3, 12] from cucumbers [4, 13], rice [5, 14] and *Arabidopsis thaliana* [6]. In particular, six putative *RAFS* genes (*AtRFS1-6*) have been identified in *Arabidopsis Thaliana* [15, 16], but only *AtRFS5* has demonstrated *RFS* activity, while *AtRFS4* has shown little raffinose synthetic capacity. Except for *AtRFS4* and *AtRFS5*, *AtRFS2* has been predicted as drought induced *RAFS* in *Arabidopsis thaliana* [17]. Based on bioinformatics methods, few maize *RAFS* genes have been identified [18]. A unique maize *RAFS* gene has been found to play a vital role in seed viability and longevity [19]. However, there is no evidence to show that over-expression of *RAFS* can increase the content of raffinose. Despite extensive research on the role of gossyps in regulating or responding to abiotic stress in plants, the role of gossyps in drought resistance in plants remains unclear.

Synthetase *RAFS* plays an important role in the plant seed acquisition of drought resistance and the extension of seed life [20, 21]. Owing to the instability of raffinose synthetase, however, there have been relatively few research reports on synthetase *RAFS*, especially systematic research on the *RAFS* gene family in *Gossypium* species [22]. Cotton is an important industrial crop in national economic production, and improving its stress resistance is the focus of current cotton cultivation research [23]. To comprehensively study the evolution and expression patterns of the *RAFS* gene superfamily in *Gossypium* species, we identified *RAFS* genes and constructed a phylogenetic tree in the present study. We also analyzed the synteny, promoters and co-expression network associated with these *RAFS* genes. The constructed phylogenetic tree showed that *RAFS* genes could be grouped into four subfamilies. Through research of the cotton *RAFS* gene family, this study provides a certain theoretical basis for further research on cotton’s stress resistance mechanism and the improvement of cotton’s stress resistance.

## Results

### Identification of *RAFS *genes in *Gossypium* species

Using 21 known *RAFS* protein sequences of Arabidopsis, rice and maize as a query for BLAST, along with a Pfam search, a total of 74 *RAFS* genes in *G. arboreum*, *G. raimondii*, *G. hirsutum* and *G. barbadense* were identified. Among them, there were 12 *G. raimondii RAFS* genes named *GrRFS1-GrRFS12*, 12 *G. arboreum RAFS* genes named *GaRFS1-GaRFS12* according to their locations on the chromosome, and 25 *G. hirsutum RAFS* genes named *GhRFS1A-GhRFS13A* and *GhRFS1D-GhRFS12D* based on their locations to the At and Dt sub-genomes, respectively. Moreover, 25 *G. barbadense RAFS* genes were named *GbRFS1A-GbRFS12A*, *GhRFS1D-GhRFS10D* and *GbRFS23-GbRFS25* within the scaffold (Additional file 1: Table S1). The prediction of subcellular localization showed that most of the 74 *RAFS* genes were localized in the periplasm and a few were in the cytoplasm and outermembrane (Additional file 1: Table S1).

### Phylogenetic analysis, chromosomal distribution and structural features of cotton *RAFS* genes

**Fig. 1:**
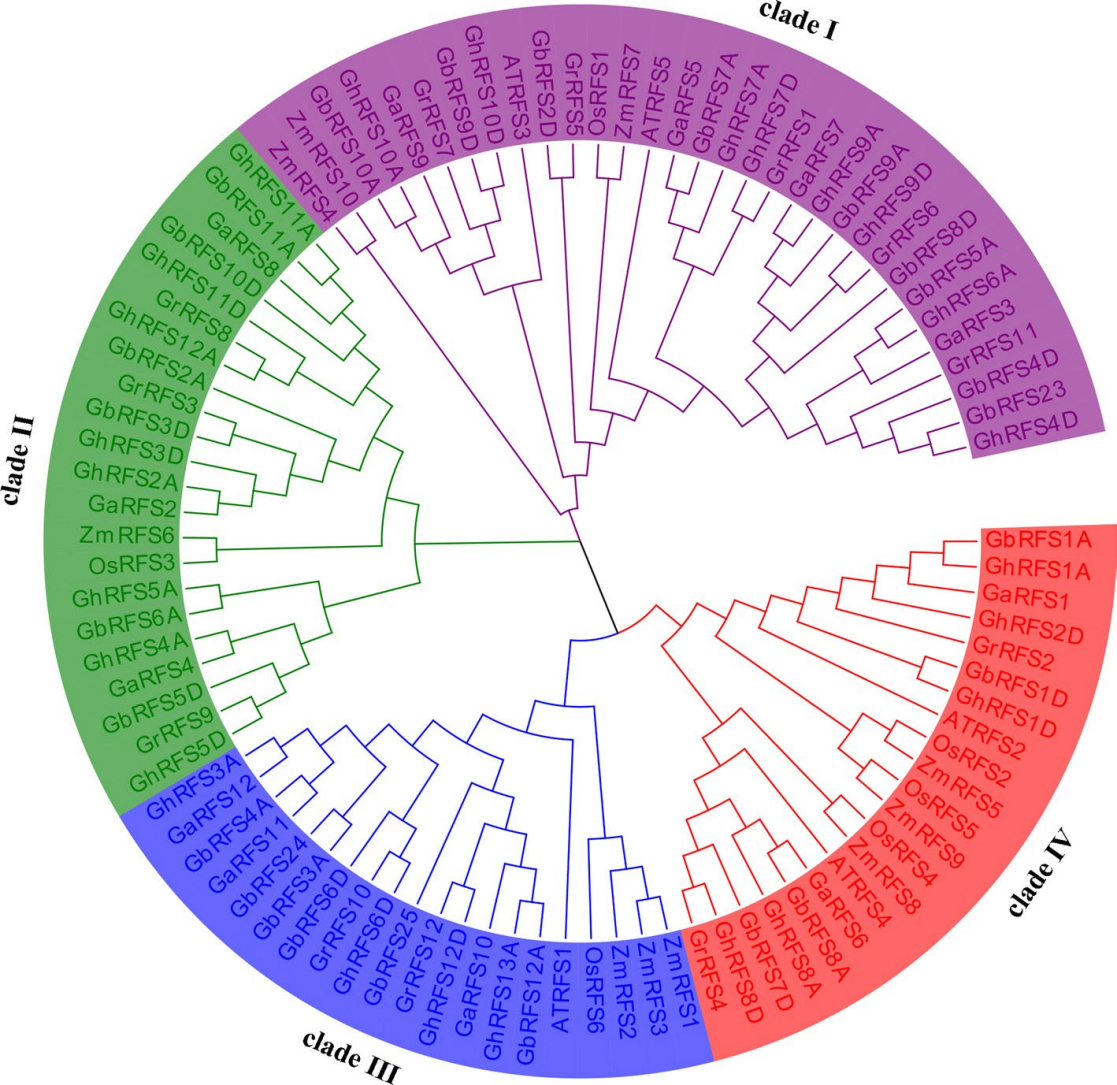
Phylogenetic trees of *RAFS* genes. The neighbor joining phylogenetic tree of 95 *RAFS* genes of the following seven species: *G. hirsutum, G. arboreum, G. barbadense, G. raimondii, A. thaliana, O. sativa* and *Z. mays.* The gene family was divided into four *RAFS* subfamilies, named clade I, II, III, and IV

To investigate the evolutionary history of the *RAFS* gene family, phylogenetic trees were constructed using all 95 full-length *RAFS* protein sequences in different species. The result showed that all of the *RAFS* genes in the Arabidopsis, rice, maize and four cotton species were divided into four clades (Fig. 1). Clade I was the largest clade, with 32 members, including five, four, nine and eight *RAFS* genes of *G. raimondii*, *G. arboreum*, *G. barbadense* and *G. hirsutum*, respectively. Clade II had 22 members, including three, three, six and eight *RAFS* genes of *G. raimondii*, *G. arboreum*, *G. barbadense* and *G. hirsutum,* respectively. Clade III had 20 members, including two, three, six and four *RAFS* genes belonging to *G. raimondii*, *G. arboreum*, *G. barbadense* and *G. hirsutum,* respectively. Clade IV had 21 members, including two, two, four and five *RAFS* genes belonging to *G. raimondii*, *G. arboreum*, *G. barbadense* and *G. hirsutum,* respectively (Fig. 1).

**Fig. 2:**
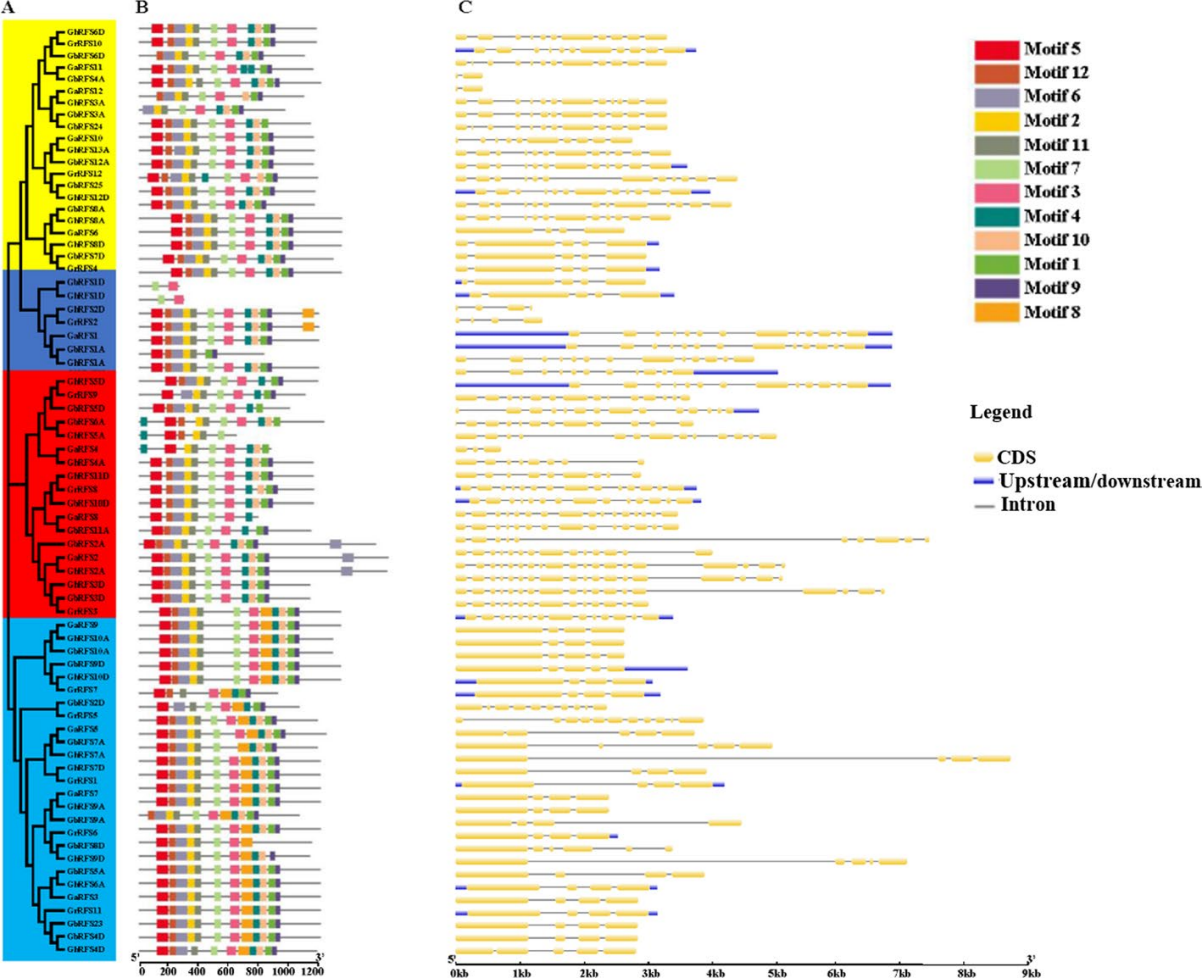
Conserved motifs and exon–intron organization of *RAFS* genes in four *Gossypium* species. **a** Neighbor joining phylogenetic tree of the *RAFS* genes in four *Gossypium* species. **b** Conserved motifs. **c** Gene structures. Yellow, blue, silver gray reperesent CDS, upstream/downstream acting elements, Intron, respectively

Combined with evolutionary tree analysis, the result of the conserved motifs of cotton *RAFS* genes showed that the motif distribution of 21 genes in group one was relatively consistent, of which all of the genes, except *GhRFS3A*, contained 11 conserved motifs with a consistent order (Fig. 2a, b). For group two, *RAFS* genes *GbRFS1D* and *GhRFS1D* only contained motif five and motif seven, while the other five genes almost contained the same motifs, showing that there were significant differences for the structure of *RAFS* genes in this group (Fig. 2a, b). Genes in group three and four had relatively consistent motif distributions. Generally, there were significant differences in the motif structure of cotton genes in the four branches. The motif distribution of genes in groups one and three was similar (Fig. 2a, b). *RAFS* genes in group four were relatively conservative. Genes in group two had the greatest variation. The difference in protein structure between different subfamilies may be the result of long-term gene genetic evolution.

The analysis of exon–intron configurations of the *RAFS* genes revealed that there were differences in the number of exons among genes of different subfamilies, but most *RAFS* genes of the same subfamily had the same exon–intron structure (Fig. 2c). *GaRFS11*, *GbRFS4*, *GbRFS1D* and *GhRFS1D* had the fewest exons, with fewer than 4, while *GrRFS2* and *GhRFS2* had 18 exons. The distribution of exons showed that *RAFS* genes clustering in the same subfamily in the phylogenetic tree usually had similar gene structures and exon numbers, which was highly conservative. However, there were also a few *RAFS* genes with different lengths and exon numbers within each subfamily.

**Fig. 3:**
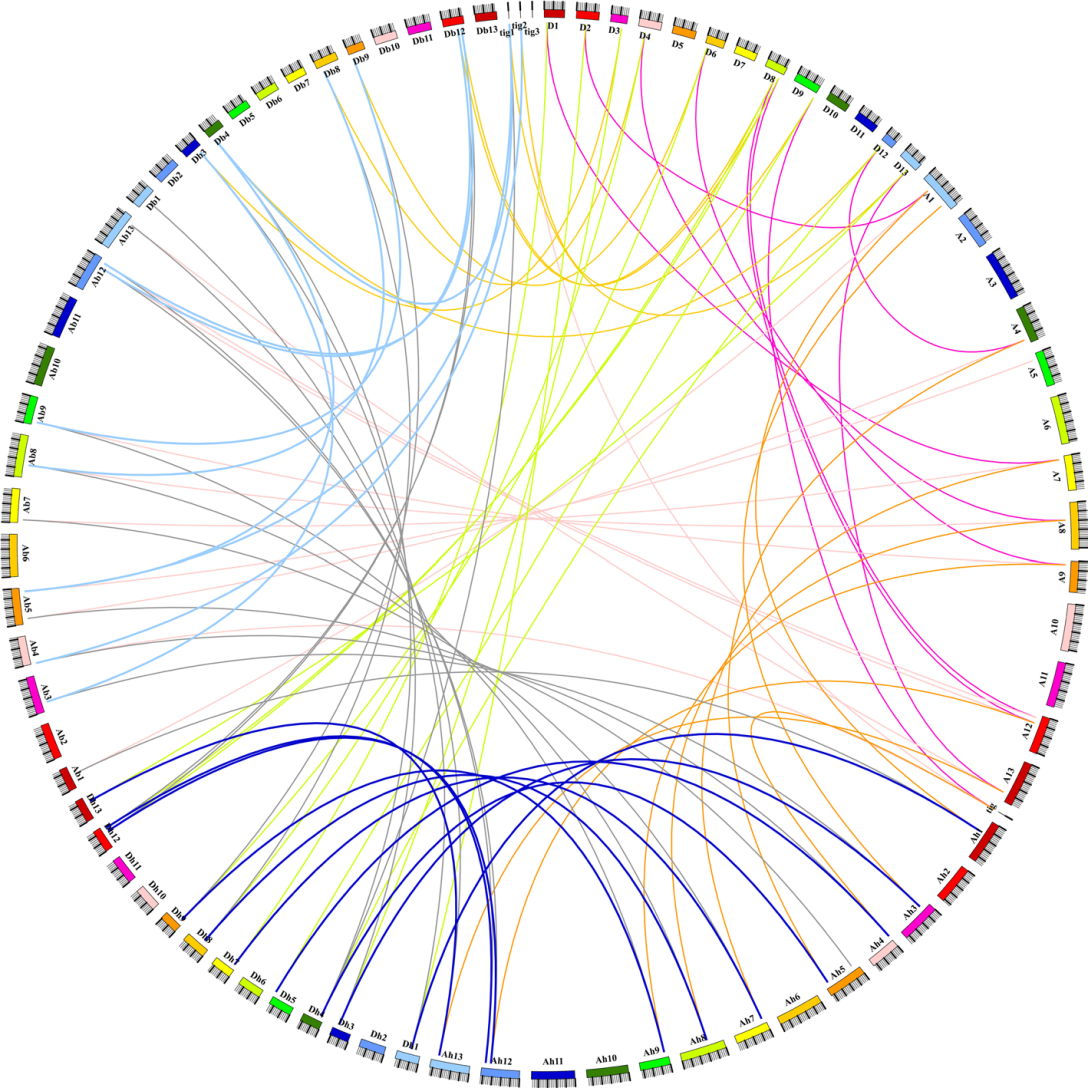
Circos map of *RAFS* homologous gene pairs among *G. arboreum*, *G. raimondii*, *G. barbadense*, and *G. hirsutum*

The chromosomal distribution of cotton *RAFS* genes showed that the ten *G. arboreum RAFS* genes were localized to the At-subgenome and two were localized to scaffolds. Moreover, 12 *G. raimondii RAFS* genes were localized to Dt-subgenome, 12 *G. barbadense RAFS* genes were localized to the At-subgenome, and ten *G. barbadense RAFS* genes were localized to the Dt-subgenome. Furthermore, three *G. barbadense RAFS* genes were localized to three scaffolds, respectively. Thirteen and 12 *G. hirsutum RAFS* genes were localized to the At and Dt-subgenomes, respectively (Additional file 2: Table S2). The syntenic relationships among these genes were visualized in circular maps (Fig. 3). A total of 74 pairs of homologous genes exhibited a collinear relationship in *G. raimondii*, *G. arboreum*, *G. barbadense* and *G. hirsutum*.

### Expression analysis of *RAFS *genes in *G. hirsutum*

**Fig. 4:**
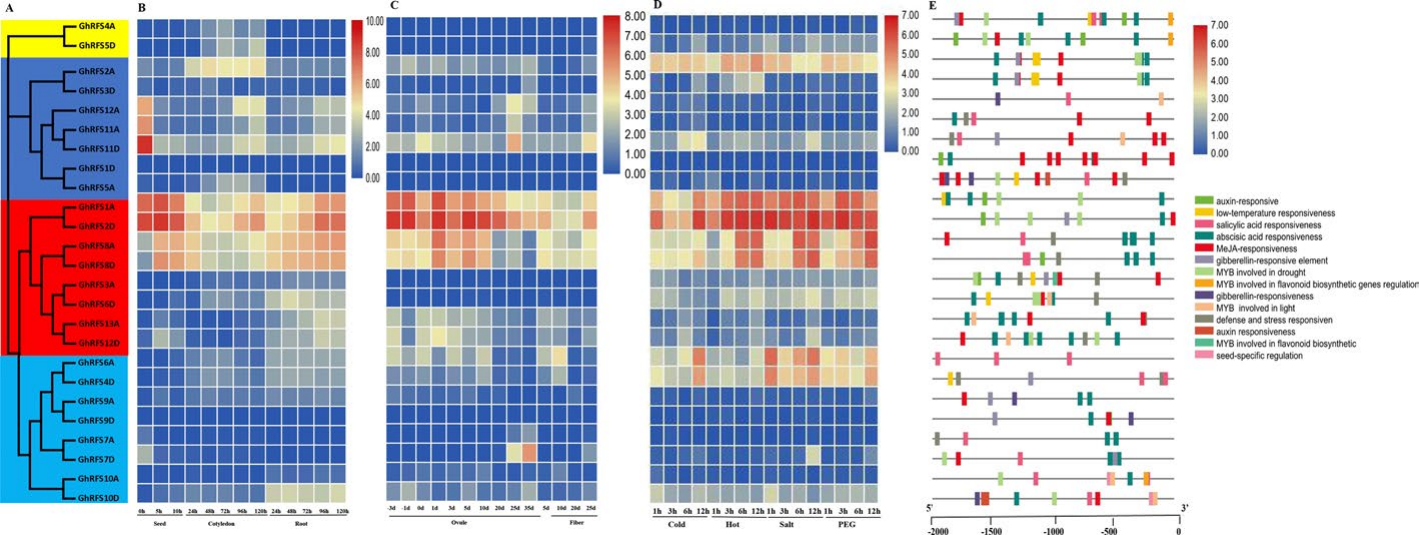
Promoters and expression analysis of GhRFS genes in G. hirsutum **a** Neighbor-joining phylogenetic tree of the GhRFS gene family in G. hirsutum.** b** Expression profile of GhRFS genes in the development process of seed, cotyledon and root.** c** Expression profile of GhRFS genes in the development process of ovule and fiber.** d** Expression profile of GhRFS genes under cold, hot, salt and PEG stress.** e** cis-elements in promoters of RAFS genes

To investigate the possible functional roles of different *RAFS* homologs in *G. hirsutum*, we analyzed the expression patterns of a total of 25 *G. hirsutum* candidate *RAFS* genes based on published RNA-seq data (Fig. 4, Additional file 3: Table S3). The expression profiles were analyzed for seeds, roots, cotyledons, ovules and fibers, as well as different time series of seeds under conditions of cold, heat, salt and drought stresses. As shown in Fig. 4b, the *GhRFS* genes in clades I and IV were almost not expressed in the development processes of seed, cotyledon and root, whereas the genes in clade II were partially expressed with *GhRFS12A*, *GhRFS11A* and *GhRFS11D* having the highest expression level in the early stage of seed germination. In clade III, *GhRFS1A*, *GhRFS2D*, *GhRFS8A* and *GhRFS8D* had the highest expression level in any of the tissues, which indicates that these four genes may play vital roles in promoting the vegetative growth of *G. hirsutum*. For the *RAFS* gene expression profile during development stages of ovule and fiber, results showed that the four genes *GhRFS9D, GhRFS7D, GhRFS8D* and *GhRFS2D* were not expressed, while the other genes were expressed (Fig. 4c). Genes in subfamilies I and IV were also not expressed, and some genes in subfamily II were expressed. For instance, *GhRFS11D* was partially expressed and had the highest expression level at 25 DPA of ovule development. In clade III, the genes *GhRFS1A* and *GhRFS2D* were expressed during ovule development, but the relative expression level decreased compared with that during the development of the seed, cotyledon and root, indicating that the genes may play a certain role in the early development stage of the ovule. Similarly, *GhRFS4A*, *GhRFS4D* and *GhRFS8A* were highly expressed in the early development stage of the ovule but were decreased at 20–35 DPA.

To further assess the potential role of *G. hirsutum RAFS* genes in response to abiotic stress processes, we analyzed gene expression patterns under different abiotic stress conditions (Fig. 4d). Genes *GhRFS1A* and *GhRFS2D* were highly expressed in all of the abiotic stress conditions. The expression levels of *GhRFS8A* and *GhRFS8D* were relatively low at early stages of abiotic stress (cold, heat, salt and drought stresses), while it sharply increased in the late stages, which suggested that these genes may be involved in responding to abiotic stresses by over-expression to increase raffinose content when subjected to abiotic stresses. Noteworthy, not all of the genes from the same clade, with similar sequences, had the same expression level and gene functions. For example, most of the other *GhRFS* genes were not expressed or lowly expressed in any of the abiotic stress conditions. Moreover, to clarify whether *cis*-acting elements influenced the expression levels, we analyzed the 2000 bp sequence upstream of the start codon of *GhRFS* gene. The results showed that the promoters of almost all *GhRFS* contained hormone-related *cis*-acting elements, such as GA_3_, except for *GhRFS6A*, *GhRFS11D* and *GhRFS6A*. In addition, *GhRFS1A*, *GhRFS2D*, *GhRFS8A* and *GhRFS8D*, expressed highly under abiotic stress conditions, specifically possessed *cis*-acting elements MYB and ANX, which proved to be relevant to abiotic stresses in plants (Fig. 4e).

### Co-expression network of *GhRFS* genes under abiotic stress

**Fig. 5:**
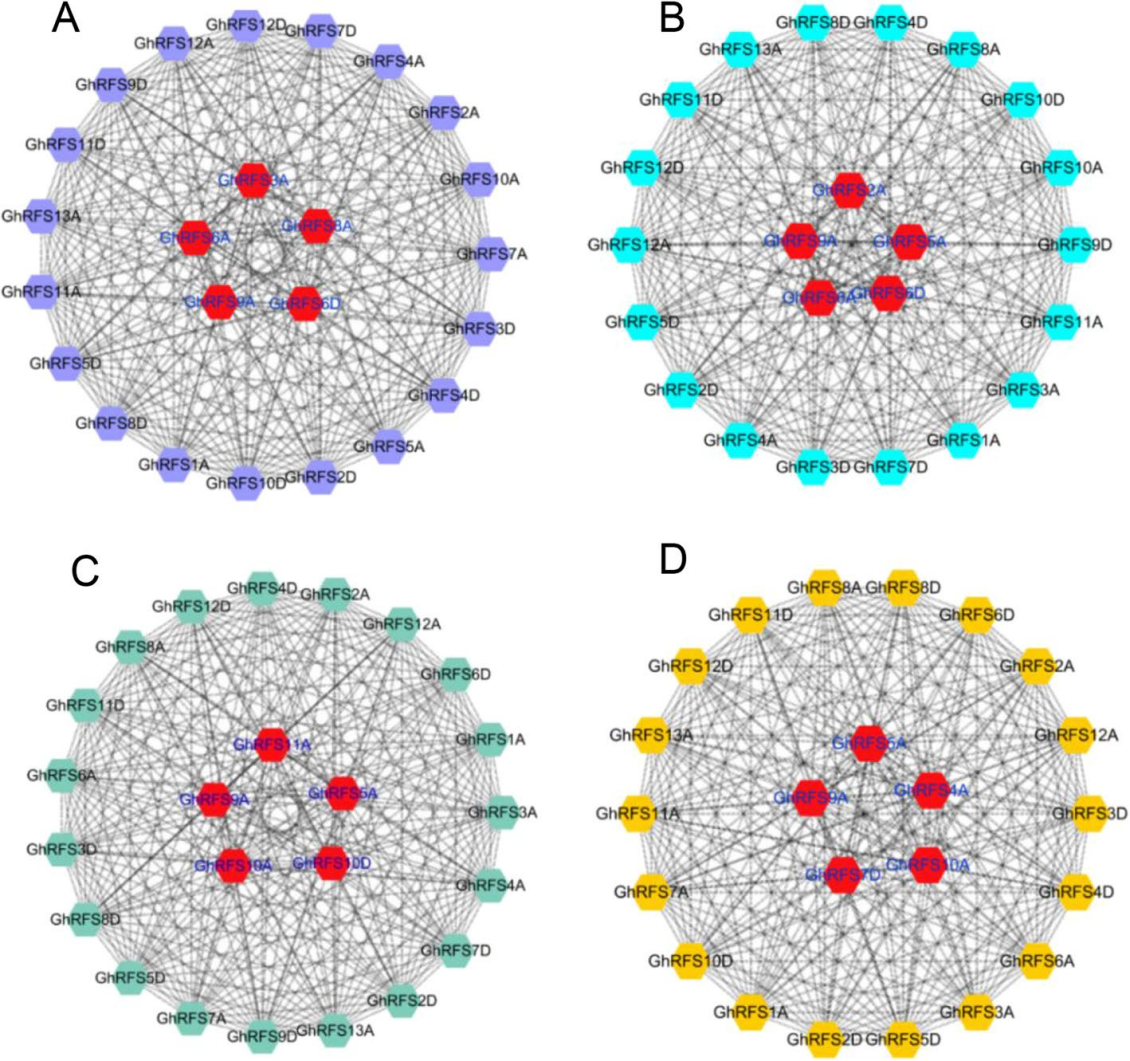
Co-expression networks of *GhRFS* genes under stress treatments. **a** Cold stress **b** Hot stress **c** Salt stress **d** PEG stress

Pearson’s correlation coefficient was frequently used to construct the gene co-expression network. In this study, the method was used to construct the co-expression networks of 25 *GhRFS* genes in *G. hirsutum* to further understand its role in abiotic stresses (Fig. 5). As a result, 166 gene pairs were positively correlated and 111 gene pairs were negatively correlated under cold stress (Fig. 5a) with hub genes *GhRFS3A*, *GhRFS6A*, *GhRFS8A*, *GhRFS9A* and *GhRFS6D*. Similarly, 177 gene pairs and 77 gene pairs were positively and negatively correlated, respectively, under heat stress with hub genes *GhRFS2A*, *GhRFS5A*, *GhRFS6A*, *GhRFS9A* and *GhRFS6D* (Fig. 5b). Under salt stress, 198 gene pairs were positive correlated and 79 gene pairs were negative correlated with hub genes *GhRFS5A*, *GhRFS9A*, *GhRFS10A*, *GhRFS11A* and *GhRFS10D* (Fig. 5c). Moreover, 134 gene pairs were positively correlated and 120 gene pairs were negatively correlated under drought stress with hub genes *GhRFS4A*, *GhRFS6A*, *GhRFS9A*, *GhRFS10A* and *GhRFS7D* (Fig. 5d). Co-expression network analysis indicated that these hub *GhRFS* genes in the network may play an important role in responding to abiotic stress of cold, heat, salt and drought for *G. hirsutum*.

## Discussion

Raffinose accumulates in higher levels in plants when responding to abiotic stress [10]. Several studies of *RAFS* have been carried out in *Arabidopsis thaliana* and *Zea mays* previously, which predicted their role as a strong drought stress resistance. In Arabidopsis, the knockout mutant involved in raffinose biosynthesis has shown no abnormalities during normal growth, except for disturbed raffinose content and reduced germination completion in the dark [3, 24, 25]. By studying the characteristics of maize knockout mutant (*ZmRFS*) and Arabidopsis plants expressing *ZmRFS*, we found that raffinose could directly and positively regulate drought stress tolerance of plants [26]. Despite extensive research, the function of raffinose in response to plant abiotic stress remains unclear, and there is no evidence that over-expression of *RAFS* genes can regulate raffinose content in vegetative tissue of in cotton, which is cultivated worldwide and faces severe biotic and abiotic stresses. Raffinose participates not only in the development of plant seeds but also in the response of plants to external stresses. With the completion of cotton genome sequencing, it is very important to study cotton *RAFS* genes from the whole genome. In this study, we performed a whole-genome identification of candidate *RAFS* genes in *G. hirsutum, G. barbadense*, *G. arboreum* and *G. raimondii*, mainly focusing on the allotetraploid cotton *G. hirsutum*, with the aim of understanding its roles, their evolutionary relationships and expression in response to various abiotic stresses. The results can provide basic information and serve as an important resource for further investigations into the candidate *RAFS* gene.

A total of 74 candidate *RAFS* genes in the genomes of *G. hirsutum, G. barbadense*, *G. arboreum* and *G. raimondii* were identified. The phylogenetic analysis revealed that all of the candidate *RAFS* genes were divided into four subfamilies, and the numbers of identified candidate *RAFS* genes in different cotton species were almost the same based on the species ploidy. That is, tetraploid cotton had about twice the number of candidate *RAFS* genes as diploid cotton, for example, only 12 genes in *G. barbadense* and *G. arboreum* as compared to 25 genes in upland cotton and *G. raimondii*, illustrating that candidate *RAFS* genes were conserved during evolution and underwent large-scale expansion in higher plants. Moreover, clade I was the least abundant member of the candidate *RAFS* clade.

Combined transcription factors and *cis*-elements upstream of the promoter regions can regulate gene transcription, which ultimately causes gene expression. We predicted that various *cis*-elements in the candidate *RAFS* gene promoter region were associated with plant hormone response elements and abiotic stress response elements (Fig. 2b). Previous studies also supported our predictions about the involvement of most *RAFS* genes in plant phytohormones responses, the most important one being abiotic stress. For example, *ZmRAFS*, a member of the raffinose gene family in maize, is a key gene that protects plants from abiotic stress. Constitutive over-expression of the *ZmRAFS* gene has been found to enhance plant drought stress tolerance without causing adverse effects to the plants under normal conditions [26]. Furthermore, manipulation of raffinose family oligosaccharides metabolic pathway can also improve the seed vigor [27]. Manipulating raffinose to enhance both plant drought stress tolerance and seed longevity has become promising in crop plants. Based on species ploidy, the even distribution of *RAFS* genes among all four species of cotton, *G. hirsutum, G. barbadense, G. arboreum* and *G. raimondii*, may have occurred because of gene tandem, segmental, or whole-genome duplication events during evolution (Fig. 3). Gene structure analysis showed that *GhRFS1D, GhRFS5A* and *GhRFS6A* were intron less frequently, and their proteins exhibited similar patterns of protein motif distribution (Fig. 2c). These structural differences in exons/introns may have been due to splicing selectivity and can be used to identify their evolutionary mechanisms [28]. Such low-intron genes can evolve rapidly through replication or reverse transcription and can then be incorporated into the genome. Genes with more introns are thought to have gained new functions in the evolutionary process [29, 30]. Limited variation in the exon/intron structure of *GhGRXs* was observed compared to other plants.

The gene expression profile of candidate *RAFS* genes in upland cotton was also determined based on RNA-seq data. For example, the FPKM values for *GhRFS12A*, *GhRFS11A* and *GhRFS11D* had the highest expression level in the early stage of seed germination, and *GhRFS1A, GhRFS2D, GhRFS8A* and *GhRFS8D* had the highest expression level in all of the tissues, which indicates that these four genes may play vital roles in promoting the vegetative growth of *G. hirsutum*; moreover, *GhRFS9D*, *GhRFS7D*, *GhRFS8D* and *GhRFS2D* were not expressed, while the other genes were expressed in the development process of the ovule and fiber (Fig. 5b).

In this study, the structure, evolution, gene location and gene co-expression network of the cotton candidate *RAFS* genes were explored. The results showed that the *RAFS* gene family contained one conserved domain and could be divided into four subfamilies across the four cotton species. Most of the genes were evenly distributed on the chromosomes of the four cotton species, and there were differences in gene expression and co-expression network during the plant development process and under abiotic stresses. This study provides some clues that clarify the role of *RAFS* genes in ovule formation and seed, root, cotyledon and fiber development of plants, as well as the specific role they play in how plants respond to abiotic stresses. It also provides a basis for further exploration of the identification of stressresistant new materials in cotton. Based on the results of this study, scientists can further confirm the role of these candidate *RAFS* genes in cotton with in vitro validation experiments.

## Conclusion

Raffinose synthetase (*RAFS*) is a key enzyme in the synthesis process of raffinose. Although *RAFS* genes of some species have been identified and functionally characterized, whether *RAFS* contributes to resistance to abiotic stress in *Gossypium* species had not been investigated. In this study, we conducted a complete genome wide survey of *RAFS* gene family in four *Gossypium* species which results into identification of 74 *RAFS* genes distributed into evolutionary conserved four groups. RNA-seq and promoter analyses revealed genes *GhRFS1A*, *GhRFS2D*, *GhRFS8A* and *GhRFS8D*, with ANX and MYB motifs, were highly expressed in plant seeds, cotyledonsroots, and under heat, cold, salt and drought stress conditions, which implied that plant hormones may enhance the expression of genes and then contribute to resist stress. These results elucidate the potential function in response to abiotic stress for *RAFS* genes in *Gossypium* species.

## Methods

### Delimitation of *RAFS *homologous genes in *Gossypium* species

Whole-genome data of all four *Gossypium* species (*G. arboreum*, *G. raimondii*, *G. hirsutum* and *G. barbadense*) used in this study were downloaded from CottonFGD database (https://cottonfgd.org/about/download.html). A local BLASTp search [31] using the known *RAFS* genes of *Arabidopsis thaliana* (*AtRAFS1-AtRAFS5*), *Oryza sativa* (*OsRAFS1-OsRAFS6*) and *Zea mays* (*ZmRAFS1-ZmRAFS10*) as a query against all of the protein sequences in each genome was implemented to obtain *RAFS* homologs with an E-value cutoff of 10^−5^. To identify raffinose synthetase family members more accurately, the profile hidden Markov model (PF05691) of the HMMER3.0 program [32] was further applied to search all of the hits with the default parameters.

### *RAFS* sequence alignment and phylogenetic tree construction

MAFFT, an accurate alignment software, was used to align multiple sequences with default parameters [33]. The neighbor joining (NJ) model in software MEGA7.0 was used to construct phylogenetic trees, and the bootstrap value was set to 1000 [34]. The phylogenetic tree was displayed and beautified using iTOL (https://itol.embl.de/). The subcellular location of the *RAFS* genes was predicted through the CELLO v.2.5 website (http://cello.life.nctu.edu.tw/) [35].

### Motif analysis and chromosome location of *RAFS* genes

Conserved functional motifs of *RAFS* genes were identified using the program Multiple Em for Motif Elicitation (MEME, http://meme-suite.org/tools/meme) with the following parameters: the width of a motif was between 6 and 50aa, and the number of motifs was no more than 20 [36]. The website GSDS2.0 (http://gsds.cbi.pku.edu.cn/) was used to draw gene structure diagrams[2]. Data for four cotton species (*G. arboreum*, *G. raimondii, G. barbadense* and *G. hirsutum*) were used to compile MAP files of *RAFS* genes, and MapInspect software was used to draw the physical location map of chromosomes.

### Promoter prediction and gene expression pattern of *RAFS *in *G. hirsutum*

For *G. hirsutum RFS* genes in all four *RFS* clades, the promoter sequences, including at least 2 kb of the upstream regions, were downloaded from the CottonFGD databases (http://cottonfgd.org). The conserved cis-regulatory elements in the promoter regions were predicted using the PlantCARE (http://bioinformatics.psb.ugent.be/webtools/plantcare/html/) and PLACE (https://www.dna.affrc.go.jp/PLACE/?action=newplace) databases. Transcriptome expression data of *RAFS* genes in *G. hirsutum* TM-1 were downloaded from the NCBI database, with project number PRJNA248163, which had three biological replications per time point for each stress condition and was normalized against the control comparison group. A gene expression heatmap was produced with TBtools software. Reads were mapped to the *G. hirsutum* genome using Hisat2 software [37]. StringTie software was used to assemble and quantify the reads [38]. Fragments per kilobase of exon per million mapped fragments (FPKM) were calculated as gene expression levels. The expression levels of *RAFS* genes in the development of seeds, fibers, roots, cotyledons and ovules under the conditions of cold, heat, salt and drought stress could reflect their response to abiotic stress.

### Synteny analysis of *RAFS *genes in *Gossypium* species

To determine whether the *RAFS* gene family expanded through tandem duplication events or segmental duplication, a collinear analysis was completed with TBtools software [39].

### Co-expression network analysis of *RAFS* genes

Transcriptome data of *G. hirsutum* under cold, heat, salt, and drought stress conditions were used to build a gene co-expression network with Pearson’s correlation coefficients (r), which were calculated using the ‘cor’ function of R package. The criteria for co-expression genes identification were set at r ≥ 0.9 or r ≤ -0.9 and *P*-values ≤ 0.05 [36]. Cytoscape 3.4.0 software (http://www.cytoscape.org/) was used to visualize the gene co-expression network with graph [40]. The hub genes within the network were identified according to the topological coefficient of each node with degree N40.

## Supplementary Information

**Additional file 1**:** Table S1**. Basic characteristic of RAFS genes in G. hirsutum, G. barbadense, G. arboreum and G. raimondii.

**Additional file 2**:** Table S2**. Chromosomal locations of RAFS genes in G. hirsutum, G. barbadense, G. arboreum and G. raimondii.

**Additional file 3**:** Table S3**. Expresstion level of RAFS genes in G. hirsutum under abiotic stress

## Data Availability

The datasets supporting the conclusions of this article are included within the article and its additional files. The transcriptome data of *G. hirsutum* were downloaded from the NCBI's (National Center for Biotechnology Information) sequence read archive (SRA) with Bioproject numbers PRJNA248163.

## Abbreviations

G.: *Gossypium*
RFOs: Raffinose family oligosaccharides
DPA: Day post anthesis
FPKM: Fragments per kilobase of exon model per million mapped fragments
